# Short-Peptide-Modified Copper Nanoclusters as a Fluorescent Probe for the Specific Detection of Ascorbic Acid

**DOI:** 10.3390/s24216974

**Published:** 2024-10-30

**Authors:** Jiataiqi Li, Xin Lan, Xingcen Liu

**Affiliations:** Key Laboratory of Colloid and Interface Chemistry of the Ministry of Education, School of Chemistry and Chemical Engineering, Shandong University, Jinan 250100, China

**Keywords:** copper nanoclusters, tripeptide, assembly, fluorescent sensing, ascorbic acid

## Abstract

Metal nanoclusters assembled using short peptides as templates exhibit significant potential for development and application in the fields of catalysis and biomedicine, owing to their distinctive electronic structure, favorable optical properties, and biocompatibility. Among them, tripeptides exhibit a simpler structure and greater flexibility, enabling them to readily co-assemble with other functional components to create novel materials with significant application value. They can be assembled with copper ions to synthesize highly efficient luminescent nanoclusters, which can serve as an effective fluorescent probe. Here, we report a method for the synthesis of copper nanoclusters (Cu NCs) using tripeptides as templates, which also act as stabilizers and reducing agents. The synthesis conditions and properties were explored and optimized. Under optimal conditions, the Cu NCs exhibit excellent stability and are strongly fluorescent. The Cu NCs can detect 0.1–1.0 μmol/L of ascorbic acid with a low detection limit of 0.075 μmol/L, demonstrating high sensitivity and offering significant application potential for the trace of ascorbic acid in various substances. It also provides new ideas for the assembly of metal nanoclusters and the construction of fluorescent probe sensing platforms.

## 1. Introduction

In recent years, nanoclusters, emerging nanomaterials, have been widely used as fluorescent probes due to their good fluorescence properties [1]. Among them, metal nanoclusters (MNCs) are close in size to the Fermi wavelength and are multifunctional nanomaterials with multiple properties lying between isolated metal atoms and metal nanoparticles. They are usually composed of several or up to hundreds of metal atoms with a diameter on the nanometer level [2]. Compared with traditional fluorophores such as semiconductor fluorophores and organic small molecule fluorescent probes, MNCs have better properties such as an ultra-fine size, high fluorescence, and good biocompatibility, thus playing an important role in environmental monitoring, catalysis, bioimaging, light-emitting devices, medicine, and other fields [3,4,5,6]. In recent years, copper nanoclusters (Cu NCs), as a substitute for noble metal nanoclusters, have attracted significant attention due to their low toxicity, high biocompatibility, and easy access to low-cost precursors [7,8]. Using Cu NCs as fluorescence probes offers high sensitivity, good reproducibility, and fast analysis speed, and they have been expertly used to detect trace metal ions and other substances [3].

In order to synthesize metal nanoclusters with a better performance quickly, simply, and greenly, a series of methods, such as direct reduction, electrochemical deposition, and template methods, have been gradually developed [9,10,11]. The most commonly used method for synthesizing nanoclusters is the bottom-up template method [12]. Through this method, metal ions are induced to metal atoms through the selected template and then aggregated into nanoclusters, and the size and shape of metal nanoclusters can be controlled. Commonly used templates include DNA, small molecules, proteins, and peptides [13,14,15]. In this article, we choose peptides as a template because of their excellent self-assembly properties. In addition to single-component self-assembly, the co-assembly of peptides and functional molecules can also produce supramolecular materials, overcoming the inherent limitations of single-component self-assembly. These co-assemblies exhibit unique physical and chemical properties, such as distinctive optical characteristics [16,17]. Compared with proteins and peptides, short-peptide-modified nanoclusters have high thermal and chemical stability [17]. In addition, short peptides also have good biocompatibility and specific biological activity, which makes the nanoclusters assembled by short peptides have certain application potential in the field of biomedicine. Short peptides have multiple active sites, enabling modification of the surface of the synthesized Cu NCs to increase their practical functions. Therefore, we chose short peptides as the template for assembly [18].

Ascorbic acid, also known as vitamin C, is a human vitamin and a very effective antioxidant and can be synthesized by plants and most animals. Ascorbic acid is closely related to human health and plays an important role in maintaining normal metabolism and homeostasis [19]. For example, a lack of ascorbic acid may lead to scurvy, cataract, cancer, cardiovascular and cerebrovascular diseases [20]. Vitamin C is mainly derived from fruits, and ascorbic acid can also be used as a medicine to prevent the production of carcinogens such as nitrosamines and to prevent certain diseases [21]. In actual samples, the ascorbic acid content is often low, and it is very easily oxidized to dehydroascorbic acid [22]. Therefore, a rapid and sensitive determination method would be highly significant for practical applications.

**Figure 1:**
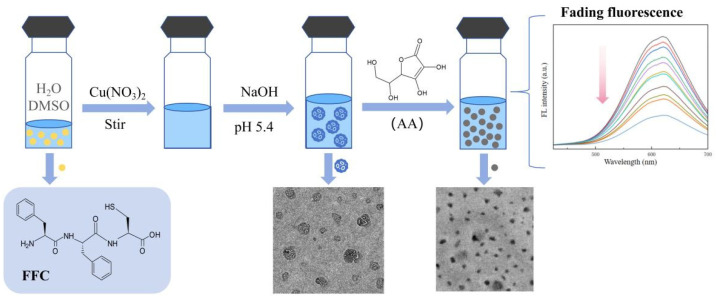
The scheme of short-peptide-modified Cu NCs as a fluorescent probe for the specific detection of ascorbic acid.

In this paper, we use a tripeptide, phenylalanine–phenylalanine–cysteine (Phe-Phe-Cys, FFC), as a template to synthesize the required Cu NCs (shown in Figure 1). The ratio between FFC and copper ions and the pH were continuously adjusted to determine the best conditions for assembly. At the same time, the stability, fluorescence lifetime, and particle size of the nanoclusters were tested to fully verify that the assembled Cu NCs perform well. Finally, the trace ascorbic acid was tested to prove that the Cu NCs have a certain application value, which can inspire the assembly and application of nanoclusters in the future.

## 2. Materials and Methods

### 2.1. Materials

All reagents and drugs used were analytically pure. The copper ions were sourced from copper nitrate in copper salt. The drug mentioned above and the sodium hydroxide solid were purchased from Guoyao Reagent Co., Ltd., Beijing, China. The tripeptide as a template came from the national peptide organism. The dimethyl sulfoxide reagent used for dissolution and the ascorbic acid used for testing were all from Aladdin Reagent Co., Ltd., Shanghai, China.

### 2.2. Instruments

In this experiment, a fluorospectrophotometer (FSP; RF-6000, SHIMADZU, Kyoto, Japan) was used to determine the fluorescence intensity of samples. A Fourier-transform infrared spectrometer (FTIR; INVENIO, Bruker Optics, Ettlingen, Germany) was used to determine the characteristic composition and structure of samples. A dynamic light scattering analyzer (DLS; Nanotrac wave II, MicrotracInc, York, PA, USA) was used to determine the hydrated particle size of the sample. A transmission electron microscope (TEM; JEM-1400, JEOL, Tokyo, Japan) can observe the morphology and particle size of samples. X-ray photoelectron spectroscopy (XPS; ESCALAB XI+, Thermo Fisher Scientific Inc., Waltham, MA, USA) was employed to detect the atomic contents of FFC and the Cu NCs.

### 2.3. The Assembly of Cu NCs

The short peptide FFC was used as a template. We fixed the copper ion concentration and adjusted the ratio of the two materials to 1:1, 2:1, 4:1, 8:1, and 16:1 by changing the concentration of the tripeptide. Then, NaOH solution (1.0 mol/L) was used to adjust the pH of each solution to 5.4, and then the fluorescence intensity of each sample was tested to determine the best ratio.

After that, we fixed the tripeptide concentration at 40 mmol/L and changed the ratio of the two materials by changing the copper ion concentration to 0.2, 1, 5, 10, 20, 100, and 500 mM. The above steps were repeated to prepare and characterize the solution, and the optimal ratio of tripeptide to copper nitrate was verified again.

This ratio was then chosen to synthesize the Cu NCs. Then, the pH of the Cu NCs was adjusted using NaOH and HCl simultaneously, and the fluorescence intensity changes in the Cu NCs were monitored to determine the most suitable pH.

The change in the fluorescence intensity of the Cu NCs solution with time was also monitored to determine whether the assembled product had a certain stability.

### 2.4. The Detection of Ascorbic Acid

The ascorbic acid solution was prepared, and the same amount of ascorbic acid was added to the Cu NCs solution each time. The fluorescence intensity was measured and compared with the original fluorescence intensity of the Cu NCs to observe quenching with the aim of measuring the detection range.

In addition, different substances, such as glucose and leucine, were tested as interfering substances. The interfering substances were mixed with the prepared Cu NCs solution, and the fluorescence intensity was measured and compared with the fluorescence intensity of the solution without interfering substances. Then, the suitability of Cu NCs for ascorbic acid detection and their anti-interference ability were verified. The whole experimental process is shown in Figure 1.

## 3. Results and Discussion

**Figure 2:**
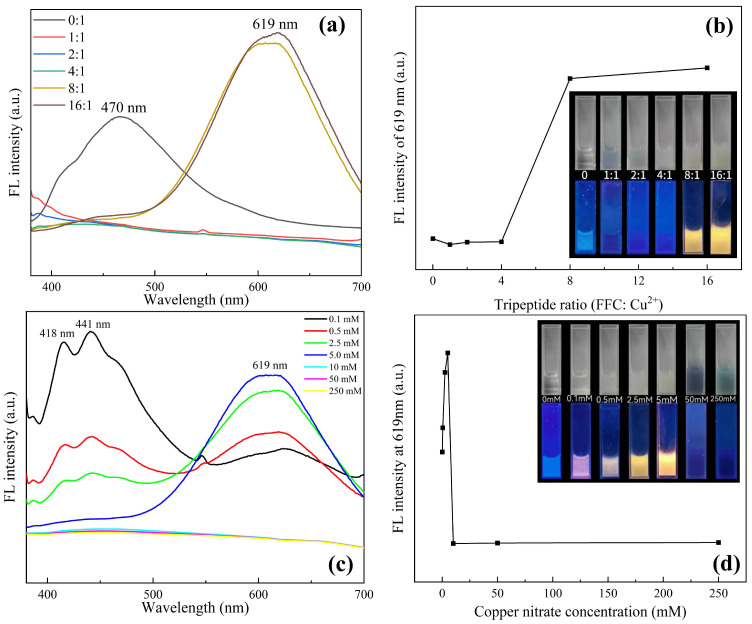
(**a**) Fluorescence spectra with different ratios of FFC and copper ions; (**b**) change in fluorescence intensity at 619 nm at various proportions corresponding to (**a**); (**c**) comparison of fluorescence spectra at different concentrations of copper nitrate with fixed FFC of 40 mM; (**d**) comparison of fluorescence intensity at 619 nm corresponding to (**c**).

As shown in Figure 2, the phenomena of the samples prepared with different proportions of initial materials under white light and a 365 nm ultraviolet (UV) lamp were compared. It can be seen from Figure 2a,b that when copper nitrate is not added, the tripeptide solution shows weak white fluorescence, and the tripeptide response wavelength is 470 nm. With the addition of copper ions, the fluorescence quenching of the tripeptide solution was observed. Because the tripeptide within cysteine is reducible, it will react with the copper ions, destroying the molecular fluorescence structure of the tripeptide. Upon increasing the concentration of tripeptide, weak orange fluorescence was observed, and with the continuous increase in tripeptide concentration, the orange fluorescence became more obvious. Meanwhile, a strong fluorescence signal at a wavelength of 619 nm shows up as the response wavelength of the Cu NCs at ratios of 8:1 and 16:1, proving that the Cu NCs were assembled successfully. As saturation was reached at the ratio of 8:1, 8:1 is determined to be the best concentration ratio for preparing Cu NCs.

From the characterization of Cu NCs prepared with different copper concentrations in Figure 2c,d, it can be seen that when the concentration of tripeptide is fixed, the fluorescence gradually changes from white to orange with the addition of copper ions, and the intensity increases. The fluorescence intensity of the Cu NCs at 619 nm also becomes stronger, while the peak of tripeptide gradually weakens until it disappears. When the concentration of copper ions in the system reaches 5 mM, the fluorescence intensity reaches a maximum. As the concentration of copper ions keeps increasing over 5 mM, there is a large number of blue or green flocculent precipitates in the solution, accompanied by the phenomenon of fluorescence quenching. This is because, during the reaction, FFC acts as a stabilizer and reducing agent to reduce copper ions. When the ratio of FFC to copper ions is 8:1, FFC can fully reduce copper ions and stabilize the synthesized Cu NCs so that they can emit strong fluorescence. When the concentration of copper ions continues to increase, FFC is not enough to reduce all copper ions in the system, and it cannot play its role as a stabilizer and may even produce some by-products. The high concentration of copper ions in the solution will make the synthesized Cu NCs agglomerate at the bottom and be adsorbed on the surface of the nanoclusters to show the blue color of copper ions [23]. It further confirms that the optimal assembly concentration of copper ions is 5 mM.

Using the optimal assembly ratio determined above, we used DLS and TEM techniques to characterize the assembled Cu NCs. The TEM image (Figure S1) shows that the obtained Cu NCs were spheres with a diameter of about 168 nm ± 30 nm, and the average size is 253 nm obtained from the normal distribution diagram obtained by DLS. The TEM results reflect the dry state of the sample at the interface; it is very sensitive to the region with high electron density, and the obtained particle size will be smaller than the actual particle size. In the DLS test, the presence of the dispersant will produce a hydrated layer around the sample particles, and the presence of larger particles in the solution may also help to increase light scattering so that the measured particle size shifts to a larger value [24]. The above will cause the particle size measured by DLS to be larger than that measured by TEM.

**Figure 3:**
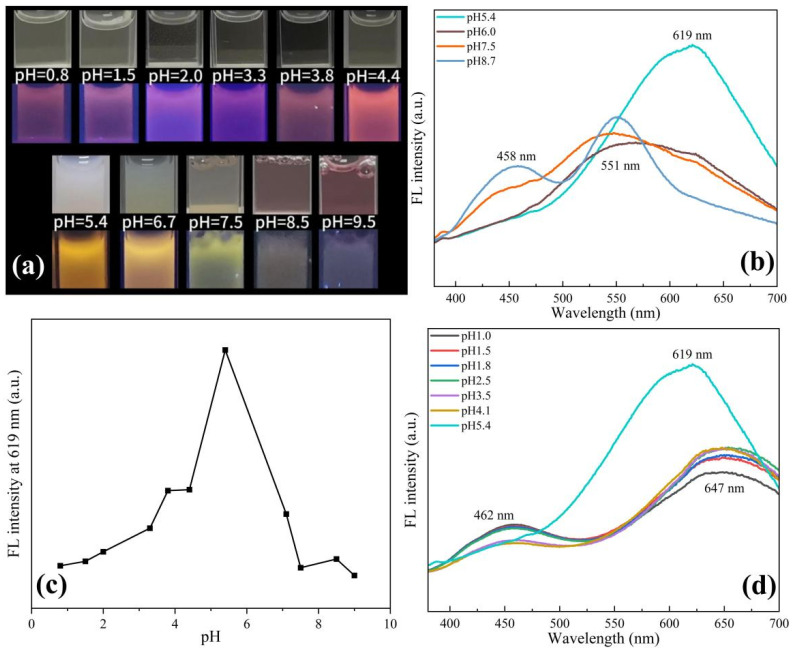
(**a**) Comparison of different pH solutions under a white light lamp and a UV lamp; (**b**) fluorescence spectra at different alkaline pH; (**c**) comparison of fluorescence intensity at 619 nm at different pH; (**d**) fluorescence spectra at different acidic pH.

Previous studies have shown that Cu NCs prepared using protein templates are very sensitive to pH changes due to structural changes [25]. Similarly, we speculate that pH changes will also have a greater impact on the assembly of Cu NCs. Therefore, we adjusted the pH of the solution to explore the optimal pH environment for the assembly of Cu NCs and also studied the effect of solution pH on Cu NCs. It can be seen from Figure 3a that the solution exhibits weak pink fluorescence under acidic conditions. As the pH increases, the fluorescence gradually becomes obvious and gradually changes to orange fluorescence. Under alkaline conditions, the solution exhibits weak yellow fluorescence. Under the white light, it can be observed that with the increase in acidity, the Cu NCs solution gradually changes from milky white and turbid to light yellow and clear solution. With the increase in alkalinity, it gradually becomes a clear pink solution. We also carried out TEM tests on the copper nanocluster solution under acidic and alkaline conditions. From Figure S2, it can be observed that under acidic and alkaline conditions, the assembled Cu NCs have different degrees of decomposition, corresponding to the clear solutions.

At the same time, we tested the fluorescence intensity of the Cu NCs solution at different pHs. As shown in Figure 3b–d, it was found that the fluorescence intensity at 619 nm was the strongest when the pH was about 5.4, which was most conducive to Cu NCs formations. At lower pH, the peak of Cu NCs redshifts to 647 nm, while the FFC peak reappears at 462 nm, revealing the partial decomposition of Cu NCs. When the pH increases to alkalinity, there is a blueshift to 551 nm, and the FFC peaks are present at 458 nm. When the pH is close to the isoelectric point of the tripeptide (pH ≈ 5.4), the tripeptide acts as a stabilizer and reducing agent to form Cu NCs, and the strong fluorescence appears due to the aggregation-induced emission (AIE) properties of metal nanoclusters. When the pH is 5.4, the appropriate aggregation of Cu NCs leads to a strong interaction between the clusters, which limits the movement of Cu NCs and reduces the chance of non-radiative paths, thus showing strong fluorescence. When the acidity or alkalinity is enhanced, the surface charge density of FFC is increased, and the aggregation of Cu NCs may be gradually decomposed and dissolved, resulting in a gradual weakening or quenching of fluorescence [26,27,28]. When the pH was adjusted back to 5.4 from acidic conditions, there was still an intense fluorescence peak at 619 nm. Then, we verified it by repeatedly adjusting the pH between 3.0 and 5.4 and measuring their fluorescence intensities. In five consecutive cycles, we obtained excellent reversibility, as shown in Figure S3. Decomposition into irregular bulk materials, combined with the determination of the above fluorescence intensity, we inferred that Cu NCs undergo a certain degree of recoverable dissociation under acidic conditions, and the assembled Cu NCs exhibit good pH-responsive aggregation and dispersion in acidic solutions. When the pH was adjusted back to 5.4 from the alkaline conditions, there was no fluorescence response at 619 nm, implying that the Cu NCs were not recoverable under alkaline conditions. Therefore, we chose 5.4 as the optimal pH for the assembly of Cu NCs.

From Figure S4, it can be found that the assembled Cu NCs are stable within a certain time range, which means FFC is fully playing its role as a stabilizer and, thus, the assembled Cu NCs have good stability.

**Figure 4:**
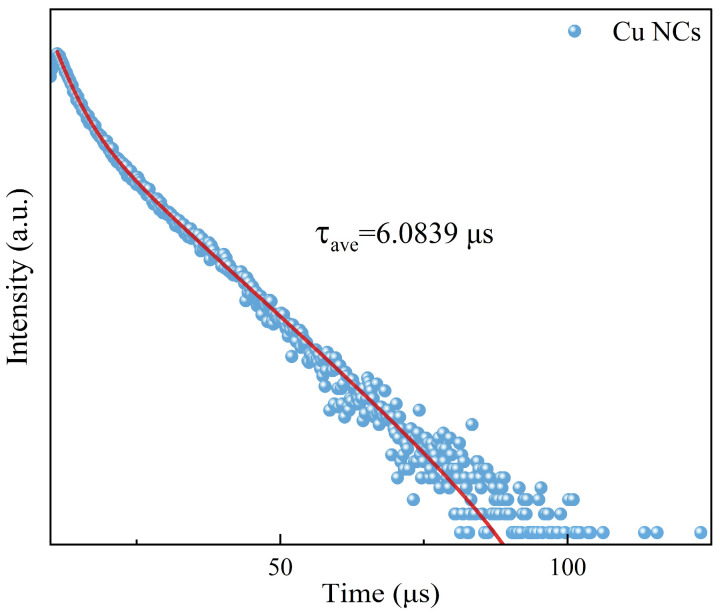
The fluorescence lifetime diagram of Cu NCs.

Combining with the data from fluorescence spectrum analysis, we set the excitation wavelength at 381 nm and the emission wavelength at 619 nm to determine the fluorescence lifetime (Figure 4) of the assembled Cu NCs (τ_ave_ = 6.0839 μs). The fluorescence lifetime directly reflects the length of time that the fluorescent molecule stays at the excited state energy level and is also directly related to the luminescence brightness and stability of the assembled material. The fluorescence lifetime of the Cu NCs is long, indicating that the material has good stability and good performance.

**Figure 5:**
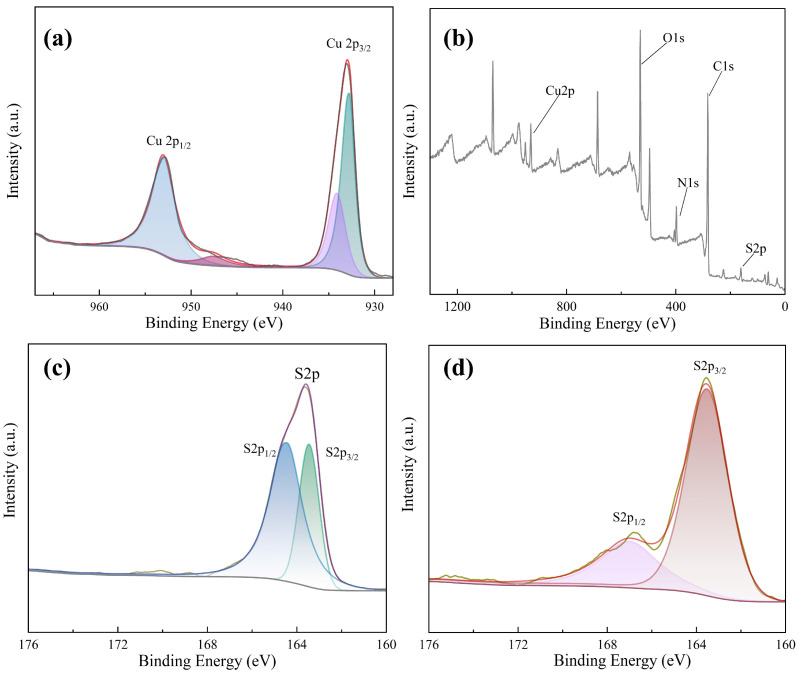
(**a**) XPS full spectrum of Cu NCs; (**b**) fine XPS spectrum of Cu elements; (**c**) fine spectrum of the S element of FFC; (**d**) fine spectrum of S element.

In order to explore the mechanism of the Cu NCs assembly, we carried out X-ray photoelectron spectroscopy (XPS) analysis. From the XPS spectrum of Cu NCs (shown in Figure 5a), the elements contained in the assembled Cu NCs are identified as Cu, O, C, S, and N elements. By observing the fine spectrum of the Cu element shown in Figure 5b, it can be seen that there are two fitting peaks at 932.77 eV and 953.02 eV, which correspond to the 2p_3/2_ and 2p_1/2_ orbitals of the zero-valent copper element, respectively. Corresponding to the standard XPS spectrum, it can be seen that the core of the assembled copper nanocluster contains Cu(0). The above results show that FFC successfully reduces divalent copper ions, enabling FFC and the reduced copper atoms to bind to each other to form the required Cu NCs.

At the same time, we also carried out an XPS analysis of FFC and focused on the comparison of the full spectrum of FFC and Cu NCs and the fine spectrum of sulfur. Comparing the fine spectra of S elements in FFC and Cu NCs (shown in Figure 5c,d), the S2p peak has very close interval spin–orbit components S2p_1/2_ and S2p_3/2_. It means that there is a spin–orbit splitting effect, and the two splitting peaks will overlap highly. The binding energy of S2p_3/2_ of FFC is 164 eV, which corresponds to the R-SH bond in FFC. In Cu NCs, the binding energy of S2p_1/2_ is 167 eV, and that of S2p_3/2_ is 163.5 eV. The overlapping degree of the two splitting peaks of the assembled Cu NCs is much smaller than that of FFC, indicating that the valence of sulfur has changed to some extent. Therefore, FFC has successfully acted as a template and reducing agent to interact with the copper in copper nitrate to synthesize Cu NCs.

**Figure 6:**
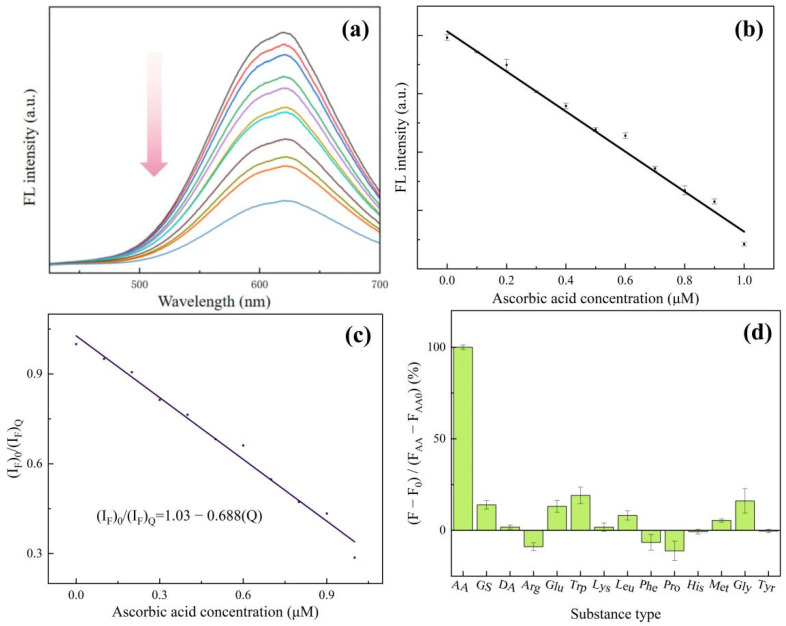
(**a**) Fluorescence spectra of Cu NCs in the presence of different ascorbic acid concentrations; (**b**) the linear relationship between fluorescence intensity and the ascorbic acid concentration; (**c**) study of static quenching mechanism of Cu NCs induced by AA; (**d**) anti-interference test of Cu NCs.

We used ascorbic acid (AA) as a substrate and used the change in fluorescence intensity to represent the catalytic ability of Cu NCs. As shown in Figure 6a,b, after the addition of AA, the fluorescence intensity of Cu NCs is greatly reduced. With the increase in AA concentration, the fluorescence emission intensity decreases steadily, but the shape and position of the fluorescence peak do not change. We can observe that the fluorescence intensity decreases with a good linear increase in concentration when the concentration of ascorbic acid is between 0.1 and 1.0 μmol/L. Therefore, the prepared Cu NCs can detect ascorbic acid in a linear range of 0.1–1.0 μmol/L, and the low detection limit is 0.075 μmol/L. Table S1 shows a comparison of AA sensors with other methods, proving our work obtained a high sensitivity with a lower LOD. Figure S5 also shows the TEM result of Cu NCs after adding ascorbic acid, demonstrating that AA could decompose the Cu NCs into small pieces, resulting in fluorescence quenching.

Fluorescence quenching is often divided into two types: dynamic quenching and static quenching. Dynamic quenching tends to shorten the fluorescence lifetime of the excited state of the substance, while static quenching has little effect on the fluorescence lifetime of the excited state of the substance. The fluorescence lifetime of the excited state of the copper nanocluster solution with ascorbic acid was measured and compared with the fluorescence lifetimes of the copper nanocluster solution without ascorbic acid. It was found that the fluorescence lifetime of the two was 6 μs, and there was no significant change. Therefore, the mechanism of AA quenching of Cu NCs was static quenching [29]. Following this, we used (I_F_)_0_/(I_F_)_Q_ to fit the curve of ascorbic acid concentration [Q], as shown in Figure 6c, and the static quenching equation was obtained as $\frac{{\left(IF\right)}_{0}}{{\left(IF\right)}_{Q}}=1$.03 − 0.688[Q], which is consistent with the general formula of the static quenching equation $\frac{{\left(IF\right)}_{0}}{{\left(IF\right)}_{Q}}=1+$ K[Q], with a complex formation constant of −0.688 [30].

In order to test the selectivity of the assembled Cu NCs for ascorbic acid detection, we studied the reaction of other substances including glucose (GS), dopamine (DA), amino acids (arginine (Arg), glutamic acid (Glu), tryptophan (Trp), lysine (Lys), leucine (Leu), phenylalanine (Phe), proline (Pro), histidine (His), methionine (Met), glycine (Gly), and tyrosine (Tyr)) with Cu NCs. By plotting the percentage of the difference in the fluorescence intensity of each substance and the difference in the fluorescence intensity due to ascorbic acid, the effect of each substance on the fluorescence intensity of Cu NCs was expressed. From Figure 6d, it can be seen that except for ascorbic acid, the fluorescence intensity of other substances on CuNCs is slightly changed. It can be concluded that the obtained Cu NCs have a good anti-interference performance and high selectivity for ascorbic acid. The assembled Cu NCs can be used to detect the trace ascorbic acid in substances.

## 4. Conclusions

We successfully prepared a copper nanocluster assembled with a short peptide as a template. The tripeptide played the role of reducing agent and stabilizer, improving the properties of Cu NCs. Then, the optimal tripeptide-to-copper-ion ratio for the assembly of the nanoclusters is 8:1, and the optimal pH range for assembly was determined to be 5.4. Various test results show that the Cu NCs have good stability and a fluorescence lifetime of 6.0839 μs. For the detection of ascorbic acid, the detection range is 0.1–1.0 μmol/L, showing that the Cu NCs can detect the lower levels of ascorbic acid in substances. They also have good selectivity for the detection of ascorbic acid. This new synthetic approach provides a reference for the construction of new sensing platforms based on nanoclusters and for the detection of ascorbic acid. It is hoped that in future research, we will continue to explore more stable and better-performing Cu NCs with peptides as templates, which can be widely used in the detection of trace substances in the fields of medicine, video, water quality, and so on, thus fully utilizing their unlimited potential as fluorescent probes.

## Data Availability

The data are contained within the article or Supplementary Materials.

## Supplementary Materials

The following supporting information can be downloaded at www.mdpi.com/article/10.3390/s24216974/s1: Figure S1: (a) TEM image of Cu NCs at pH 5.4; (b) particle size distribution of copper nanoclusters. Figure S2: (a) TEM images under pH 10.3; (b) TEM images under pH 2.8. Figure S3: The reversibility of the fluorescence intensity of Cu NCs in the pH range of about 3.0 to 5.4 during continuous cycles. For an excitation wavelength of about 381 nm, the maximum fluorescence emission intensity is monitored at about 619 nm. Figure S4: Fluorescence intensity of 619 nm change curve with time. Figure S5: TEM image of Cu NCs with ascorbic acid. Table S1: Comparison of AA sensors with other methods [31,32,33,34,35,36,37,38].
