# Supplementary material for: Short-Peptide-Modified Copper Nanoclusters as a Fluorescent Probe for the Specific Detection of Ascorbic Acid
Source: Sensors (Basel). 2024 Oct 30;24(21):6974. doi: 10.3390/s24216974 (PMC11548526; doi:10.3390/s24216974)
Supplement: Supplementary file 1 [file sensors-24-06974-s001.zip › sensors-3272102-supplementary.pdf]

supplementary materials

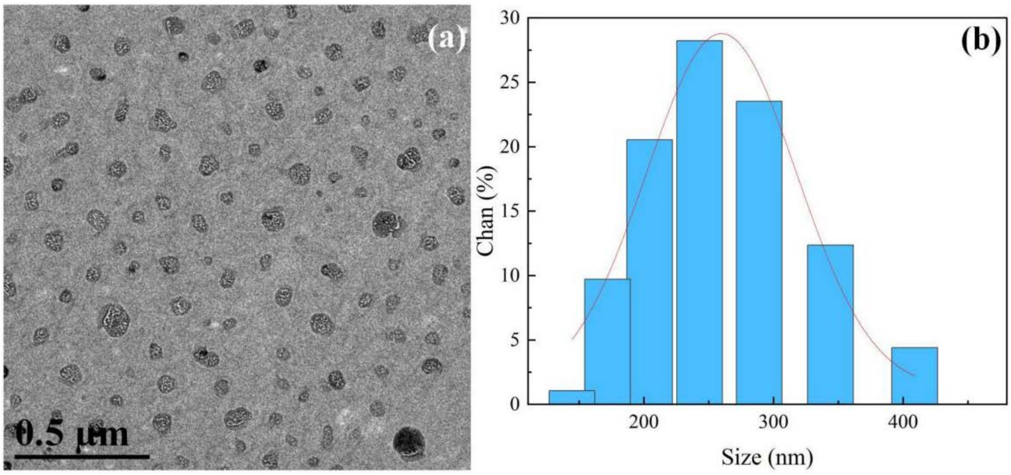

Figure S1. (a) TEM image of Cu NCs at pH 5.4; (b) Particle size distribution of copper nanoclusters.

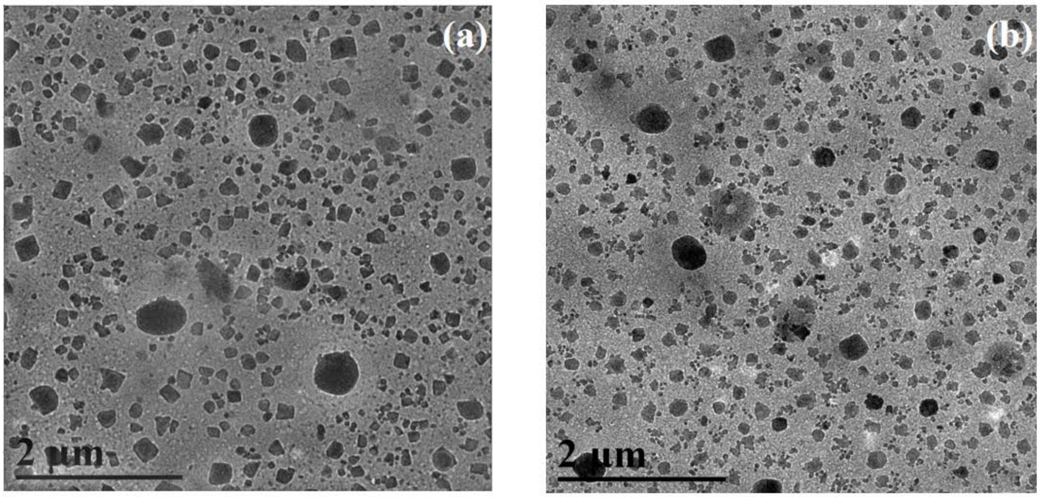

Figure S2. (a) TEM image under pH 10.3; (b) TEM image under pH 2.8.

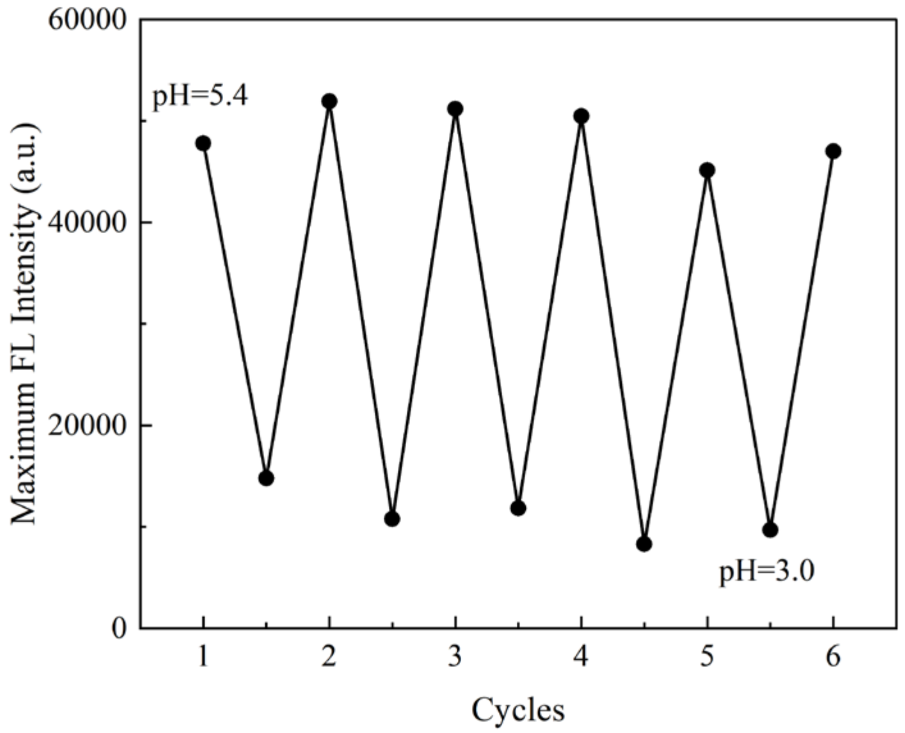

Figure S3. The reversibility of the fluorescence intensity of Cu NCs in the pH range of about 3.0 to 5.4 during continuous cycles. For an excitation wavelength of about 381 nm, the maximum fluorescence emission intensity is monitored at about 619 nm.

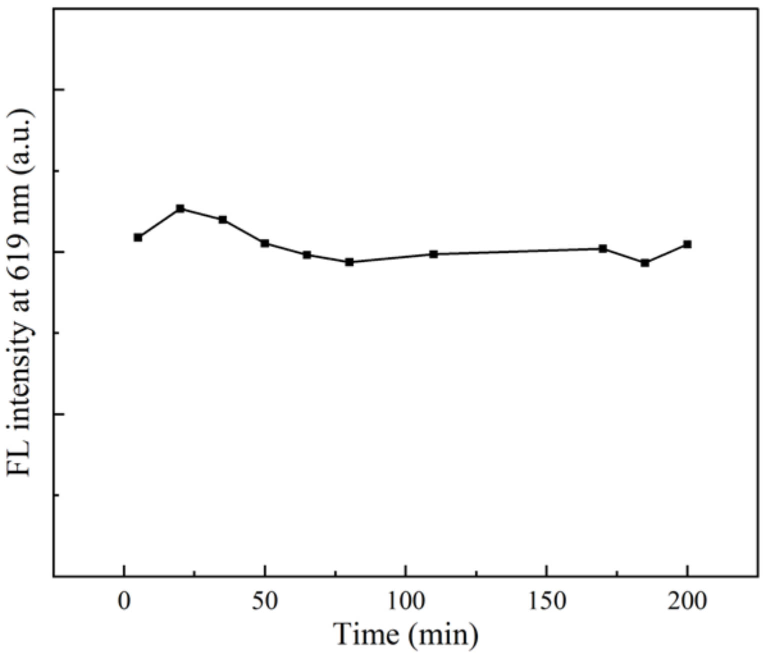

Figure S4. Fluorescence intensity of 619 nm change curve with time.

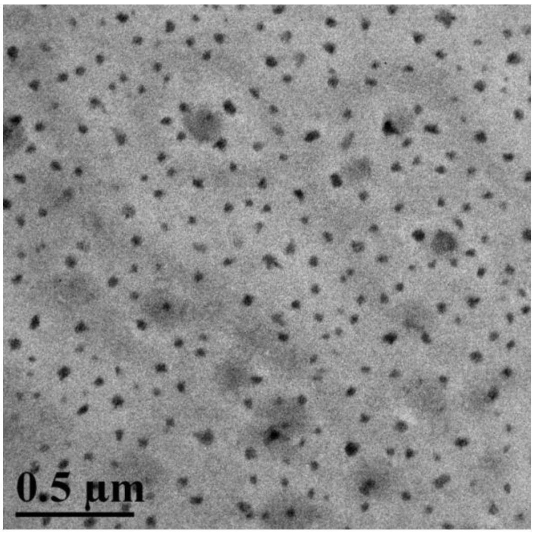

Figure S5. TEM image of Cu NCs with ascorbic acid.

Table S1. Comparison of AA sensors with other methods.

Method	Active material	Linear range（µM）	Detection limit (µM)	Reference
Colorimetry	Fe(II)-1,2ortho-phenanthroline complex	5.68-340.91	2.95	[1]
	Pd-Pt-Ir	25–800	11.7	[2]
Electrochemistry	Cu HC	5-40	1.66	[3]
	MWCNTs	18.72-1850	0.18	[4]
Fluorescence	LDH-GQDs	5-300	1.72	[5]
	F-NCDs	0-1000	2.6	[6]
	N, S-co-CDs	0-150	2.31	[7]
	Y-CDs/Cr-MOF@MIPs	25-425	10	[8]
	FFC-modified Cu NCs	0.1-1	0.075	This work

References

[1] Porto, I.S.A.; Santos Neto, J.H.; Santos, L.O. D.; Gomes, A.A.; Ferreira, S.L.C. Determination of ascorbic acid in natural fruit juices using digital image colorimetry. *Microchemical Journal*, **2019**, *149*: 104031.

[2] He, J.; He, D.X.; Yang, L.; Wu, G.L.; Tian, J.M.; Liu, Y.; Wang, W.G. Preparation of urchin-like Pd-Pt-Ir nanozymes and their application for the detection of ascorbic acid and hydrogen peroxide. *Materials Letters*, **2022**, *314*: 131851.

[3] Fernandes, D.S.; Carmo, D.R. Silsesquioxane Modified with PAMAM Dendrimer and a Bimetallic Complex for Electrochemical Detection of Ascorbic Acid. *Electroanalysis*, **2021**, *33*: 365–374.

[4] Huang, D.Q.; Li, X.; Chen, M.M.; Rui, R.; Wang, R.;Fan, S.H.; Wu, H. An electrochemical sensor based on a porphyrin dye-functionalized multi-walled carbon nanotubes hybrid for the sensitive determination of ascorbic acid. Electroanalytical Chemistry, **2019**, *841*: 101-106.

[5] Shi, H.; Chen, L. G.; Niu, N. An off-on fluorescent probe based on graphene quantum dots intercalated hydrotalcite for determination of ascorbic acid and phytase. *Sensors and Actuators B: Chemical*, **2021**, *345*: 130353.
[6] Huang,D.; Qi, H.Y.; Jing J.; Sami, R.; Jing, T.; Alsufyani, S.J.; Benajiba, N.;Madkhali, N. A continuously tunable full-color emission nitrogen-doped carbon dots and for ultrasensitive and highly selective detection of ascorbic acid. *Nanomaterials (Basel)*, **2022**, *12*: 693.
[7] Xu, S. F.; Ye, S. Q.; Xu, Y. H.; Liu, F. F.; Zhou, Y. S.; Yang, Q.;Peng, H. L.; Xiong, H. and Zhang, Z. Microwave-assisted synthesis of N, S-co-carbon dots as switch-on fluorescent sensor for rapid and sensitive detection of ascorbic acid in processed fruit juice. *Analytical Sciences*, **2020**, *36:* 353–360.
[8] Pirot, S.M.; Omer, K.M.; Alshatteri, A.H.; Ali, G.K.; Shatery, O.B.A. Dual-template molecularly surface imprinted polymer on fluorescent metal-organic frameworks functionalized with carbon dots for ascorbic acid and uric acid detection. *Spectrochimica Acta Part A: Molecular and Biomolecular Spectroscopy*, **2023**, *291*: 122340.
